# Atrial Fibrillation and Cognitive Decline: Mechanisms, Evidence, and Preventive Strategies—A Narrative Review

**DOI:** 10.3390/jcm15051899

**Published:** 2026-03-02

**Authors:** Dania Hasanein, Daniel Florin Lighezan, Oana Elena Țunea, Valentina Gabriela Ciobotaru, Norina Simona Bașa

**Affiliations:** 1 Department of Advanced Cardiology and Hemostaseology Research, “Victor Babeș” University of Medicine and Pharmacy, Eftimie Murgu Square No. 2, 300041 Timișoara, Romania; dania.hasanein@umft.ro (D.H.); dlighezan@umft.ro (D.F.L.); 2 Department of Medical Semiology I, “Victor Babeș” University of Medicine and Pharmacy, Eftimie Murgu Square No. 2, 300041 Timișoara, Romania; ciobotaru.valentina@umft.ro (V.G.C.); simona.basa@umft.ro (N.S.B.)

**Keywords:** Alzheimer’s disease, anticoagulation, atrial fibrillation, cognitive decline, dementia, direct oral anticoagulants, cerebral hypoperfusion, silent cerebral infarction

## Abstract

Atrial fibrillation (AF) is the most common sustained cardiac arrhythmia and is increasingly recognized as a risk factor for cognitive decline and dementia, independent of clinically apparent stroke. This narrative review synthesizes current evidence on pathophysiological mechanisms linking AF to cognitive decline, including cerebral hypoperfusion, silent cerebral infarction, microembolism, systemic inflammation, and shared vascular risk factors. A structured literature search was conducted in PubMed and ScienceDirect from January 2000 to October 2025, with evidence quality assessed using adapted Newcastle–Ottawa Scale criteria. Observational evidence suggests that oral anticoagulation, particularly with direct oral anticoagulants (DOACs), may be associated with reduced dementia risk compared to no treatment or vitamin K antagonists. However, most intervention studies were not designed with cognitive endpoints as primary outcomes, limiting causal inference. Current evidence supports comprehensive AF management, including guideline-directed anticoagulation, appropriate rhythm or rate control, and aggressive modification of shared risk factors. Atrial fibrillation is consistently associated with increased risk of cognitive decline and dementia through multiple interrelated mechanisms; however, randomized trials with cognitive endpoints are needed to establish causality.

## 1. Introduction

Atrial fibrillation (AF) is the most prevalent sustained cardiac arrhythmia encountered in clinical practice, affecting approximately 33 million individuals worldwide and increasing in prevalence with advancing age [1]. The characteristic irregular and chaotic atrial activity in AF arises from multiple ectopic foci generating disorganized electrical impulses within the atria. Clinically, AF may manifest with symptoms such as palpitations, dyspnea, fatigue, or dizziness, though a substantial proportion of patients remain asymptomatic [2].

Beyond its well-established role as a risk factor for stroke and systemic embolism, emerging evidence suggests that AF is independently associated with cognitive decline and dementia, even in the absence of clinically apparent cerebrovascular events [3,4,5]. A recent meta-analysis of 2.8 million individuals demonstrated that AF confers an approximately 1.4- to 1.6-fold increased risk of cognitive impairment and dementia [1]. This association persists after adjustment for traditional cardiovascular risk factors, suggesting mechanisms beyond stroke-mediated cognitive damage.

In the aging population, both AF and dementia are highly prevalent conditions that frequently coexist, sharing overlapping vascular and metabolic risk factors including hypertension, diabetes mellitus, obesity, and heart failure [6]. Cognitive deterioration in individuals with AF typically progresses through impairments in one or more cognitive domains, including attention, memory, language, executive function, and visuospatial ability. When these deficits become severe enough to interfere with daily activities, the condition fulfills diagnostic criteria for dementia [7].

The principal types of dementia include Alzheimer’s disease (AD), vascular dementia, and mixed dementia. While vascular dementia has clear mechanistic links to AF through cerebrovascular disease, the relationship between AF and AD—a neurodegenerative disorder characterized by neuronal and synaptic loss—is less straightforward and represents an area of active investigation [8]. Understanding whether AF contributes to AD pathophysiology or whether both conditions share common upstream risk factors has important implications for prevention strategies.

We conducted a structured literature search to inform this narrative review, which aims to: (1) synthesize current evidence on the epidemiological association between AF and cognitive decline; (2) present an integrated conceptual framework of the pathophysiological mechanisms potentially linking these conditions; (3) critically evaluate the evidence for preventive and therapeutic strategies; and (4) identify knowledge gaps and directions for future research.

The scientific contribution of this review lies in integrating epidemiological evidence, mechanistic insights, and therapeutic implications into a unified cardio-cerebral syndrome framework. By synthesizing data on thromboembolism, cerebral hypoperfusion, inflammation, and AF burden, this review proposes a structured conceptual model linking atrial fibrillation to cognitive decline and highlights implications for clinical management and future research.

## 2. Literature Search and Study Selection

### 2.1. Search Strategy

To ensure comprehensive coverage of the topic, we performed a structured literature search in PubMed and ScienceDirect covering January 2000 to October 2025. The search was completed on 15 November 2025.

The following search string was used in PubMed: ((“atrial fibrillation” [MeSH Terms] OR “atrial fibrillation” [Title/Abstract]) AND (“cognitive dysfunction” [MeSH Terms] OR “dementia” [MeSH Terms] OR “Alzheimer disease” [MeSH Terms] OR “cognitive decline” [Title/Abstract] OR “cognitive impairment” [Title/Abstract])) AND (“prevention” [Title/Abstract] OR “anticoagulant” [Title/Abstract] OR “mechanism” [Title/Abstract] OR “pathophysiology” [Title/Abstract]). A parallel search in ScienceDirect used equivalent terms.

### 2.2. Inclusion and Exclusion Criteria

Studies were included if they: (1) examined the association between AF and cognitive decline, dementia, or Alzheimer’s disease; (2) investigated pathophysiological mechanisms linking AF to cognitive impairment; or (3) evaluated preventive or therapeutic strategies including anticoagulation, rhythm/rate control, left atrial appendage occlusion, or risk factor modification. Eligible study designs included randomized controlled trials, prospective and retrospective cohort studies, case–control studies, systematic reviews, and meta-analyses.

Exclusion criteria comprised: (1) non-English language publications; (2) case reports and case series with fewer than 10 participants; (3) conference abstracts without full-text availability; (4) articles without full-text access; (5) studies focusing exclusively on post-stroke cognitive impairment without examining AF-specific contributions; and (6) animal studies without human validation data.

### 2.3. Study Selection and Data Extraction

**Figure 1:**
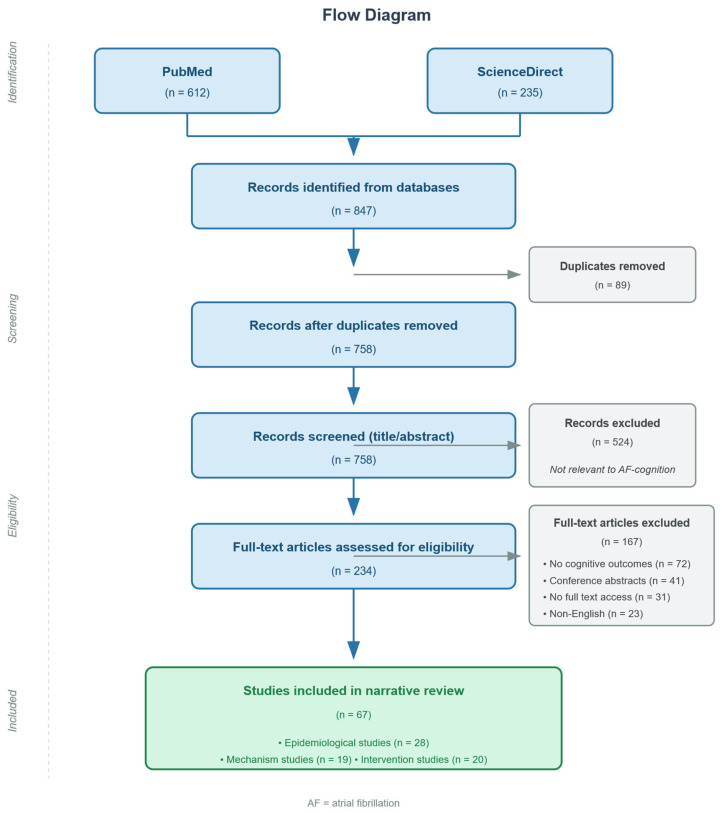
Flow diagram illustrating the study selection process. The initial database search yielded 847 records (PubMed: 612; ScienceDirect: 235). After removing 89 duplicates, 758 records underwent title and abstract screening. Of these, 234 articles were retrieved for full-text review. Following application of inclusion and exclusion criteria, 67 articles were included in the final synthesis, comprising 28 epidemiological studies, 19 mechanism studies, and 20 intervention studies. AF = atrial fibrillation.

The initial database search yielded 847 records (PubMed: 612; ScienceDirect: 235). After removing 89 duplicates, 758 records underwent title and abstract screening. Of these, 234 articles were retrieved for full-text review. Following the application of inclusion and exclusion criteria, 67 articles were included in the final synthesis. The selection process is illustrated in Figure 1.

### 2.4. Quality Assessment

To contextualize the strength of the evidence, the methodological rigor of key observational studies was considered using domains derived from the Newcastle–Ottawa Scale (selection, comparability, and outcome assessment). This appraisal was used to inform qualitative interpretation of findings rather than to generate pooled quality scores. Systematic reviews were evaluated for methodological robustness based on AMSTAR-2 domains.

Study quality informed the narrative weighting of evidence, with greater emphasis placed on large longitudinal cohorts, meta-analyses, and studies with objective neuroimaging or cognitive assessments when drawing conclusions.

## 3. Mechanisms Linking AF to Cognitive Decline

**Figure 2:**
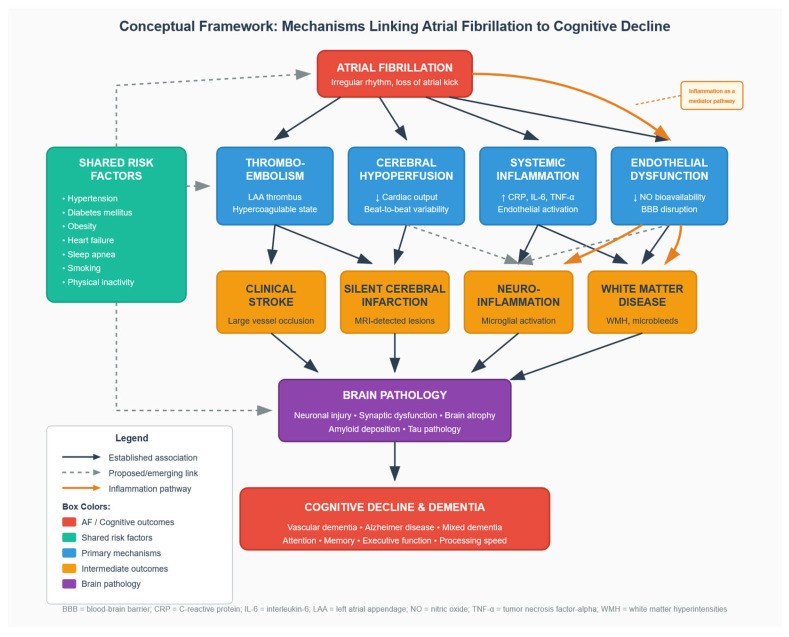
Conceptual framework illustrating the proposed pathophysiological mechanisms linking atrial fibrillation to cognitive decline. Solid black arrows indicate well-established associations; dashed arrows indicate proposed but less well-established links; solid orange arrows indicate inflammation as a mediator pathway. AF = atrial fibrillation; BBB = blood–brain barrier; CRP = C-reactive protein; IL-6 = interleukin-6; LAA = left atrial appendage; NO = nitric oxide; TNF-α = tumor necrosis factor-alpha; WMH = white matter hyperintensities.

Multiple pathophysiological mechanisms have been proposed to explain the association between AF and cognitive decline. These mechanisms are not mutually exclusive but rather appear to operate synergistically, creating a complex interplay between cardiac dysfunction and cerebral injury. Figure 2 illustrates our proposed conceptual framework integrating these mechanisms.

### 3.1. Thromboembolism and Silent Cerebral Infarction

The most direct mechanism linking AF to cognitive decline involves thromboembolic cerebrovascular events. Approximately one-third of all ischemic strokes are attributed to AF, and due to their cardioembolic origin, these events tend to be larger and more severe than strokes from other causes [9]. Meta-analytic data indicate that AF-related strokes have a 30-day mortality rate of approximately 25%, compared to 14% for non-AF strokes, and survivors experience greater residual disability [9]. The hemodynamic alterations inherent to AF—including atrial blood stasis, structural remodeling with fibrotic infiltration, atrial dilatation, and endothelial dysfunction—collectively contribute to a prothrombotic state in affected patients [10].

Importantly, a substantial proportion of AF-related cerebral injury occurs through silent cerebral infarction (SCI)—radiologically evident cerebral infarcts without corresponding clinical symptoms. Pooled data from neuroimaging studies demonstrate that SCI prevalence in AF patients ranges from 22% to 40%, compared to 10–15% in age-matched controls without AF, yielding an odds ratio of approximately 2.6 (95% CI: 1.9–3.5) [11]. A systematic review of 11 studies comprising 5317 participants found that AF was associated with a 2.4-fold increased risk of SCI (95% CI: 1.7–3.3), with higher risk observed in patients with longer AF duration and higher CHA_2_DS_2_-VASc scores [11]. Moreover, procedure-related SCI occurs in 10–50% of patients undergoing catheter ablation, with rates varying by ablation technique and cerebral MRI timing [12]. The cumulative burden of these silent infarcts—even microinfarcts <3 mm in diameter—may contribute substantially to cognitive decline over time through progressive white matter disruption and strategic infarction of cognitive networks.

### 3.2. Cerebral Hypoperfusion

AF may impair cerebral perfusion through multiple hemodynamic effects. The irregular ventricular response characteristic of AF results in beat-to-beat variability in stroke volume and cardiac output, potentially causing fluctuating cerebral blood flow. Additionally, loss of effective atrial contraction reduces ventricular filling, which may decrease cardiac output by 15–25% [2,13]. Quantitative perfusion studies using arterial spin labeling MRI have demonstrated that AF patients exhibit 10–15% lower global cerebral blood flow compared to matched controls in sinus rhythm (mean difference: −5.2 mL/100 g/min; 95% CI: −7.8 to −2.6) [14]. This hypoperfusion appears most pronounced in watershed regions and the hippocampus, areas particularly vulnerable to ischemic injury [15].

Computational modeling studies have provided mechanistic insights into AF-related cerebral hemodynamics. Beat-to-beat variability models demonstrate that the irregular R-R intervals characteristic of AF generate transient hypoperfusion episodes lasting 2–4 s, with cerebral blood flow dropping below autoregulatory thresholds in 15–20% of cardiac cycles [16]. Importantly, cerebral autoregulation—the intrinsic ability of cerebral vessels to maintain constant perfusion despite fluctuating systemic pressure—appears impaired in AF patients. Studies using transfer function analysis have shown that the dynamic cerebral autoregulation index is reduced by approximately 30% in AF compared to sinus rhythm, suggesting diminished cerebrovascular reserve [17]. This autoregulatory impairment may render the brain particularly vulnerable to hypotensive episodes and beat-to-beat variability.

Evidence supporting the clinical relevance of this mechanism comes from studies demonstrating that restoration of sinus rhythm through cardioversion or ablation is associated with improvements in cerebral perfusion. In a small prospective study, restoration of sinus rhythm after cardioversion was associated with significant improvement in cerebral blood flow compared to the AF state [18]. Whether these acute hemodynamic improvements translate to preserved cognitive function over time requires further investigation in prospective studies with long-term follow-up.

### 3.3. Systemic and Neuroinflammation

Both atrial fibrillation and dementia are characterized by proinflammatory states, suggesting that inflammation may represent a shared and mechanistically relevant pathway linking cardiac and cerebral pathology. AF promotes systemic inflammation through atrial cardiomyopathy, endothelial injury, and immune cell infiltration within the atrial wall, contributing to both electrical instability and a prothrombotic phenotype. Quantitatively, patients with AF demonstrate circulating C-reactive protein (CRP) levels approximately 1.5–2.5 times higher than matched controls (pooled mean difference: 1.8 mg/L; 95% CI: 1.2–2.4), with similar elevations in interleukin-6 (IL-6) and tumor necrosis factor-α (TNF-α) [19]. Meta-regression analyses further suggest that each 1 mg/L increase in CRP is associated with an approximately 8% higher risk of AF recurrence after cardioversion, underscoring the biological relevance of inflammatory signaling in AF substrate maintenance [20].

Beyond serving as a parallel association, inflammation may function as a mechanistic mediator linking atrial pathology to cerebral injury. Inflammatory remodeling of the atrial myocardium promotes endothelial activation and a procoagulant vascular surface, facilitating microthrombus formation and microembolism independent of clinically apparent stroke. Systemic inflammatory signaling also induces endothelial dysfunction characterized by reduced nitric oxide bioavailability, increased expression of adhesion molecules, and impaired vasoreactivity. This endothelial dysfunction represents a critical translation layer through which atrial disease is conveyed to the cerebral microvasculature, promoting blood–brain barrier disruption and vulnerability to cerebral small-vessel disease.

At the cerebral level, systemic inflammation may promote neurodegeneration through several converging mechanisms, including blood–brain barrier permeability allowing peripheral cytokines to access the central nervous system, activation and priming of microglia and astrocytes, and direct neurotoxic effects of circulating inflammatory mediators [21]. Neuroimaging studies have demonstrated that elevated inflammatory markers in patients with AF correlate with increased white matter hyperintensity burden (r = 0.34, *p* < 0.01) and reduced hippocampal volume, supporting a link between inflammation and structural brain injury [22]. Inflammatory suppression of endothelial nitric oxide synthase activity may further exacerbate both vascular dysfunction and impaired neuroprotection.

Importantly, systemic inflammation may operate both as a shared upstream confounder, reflecting common vascular risk factors, and as an AF-driven mediator that amplifies cerebral vulnerability once atrial cardiomyopathy is established. Distinguishing these roles is essential for causal inference and for identifying inflammation-targeted strategies to mitigate cognitive decline in patients with atrial fibrillation.

Emerging evidence suggests that epigenetic regulation acts as an upstream amplifier of inflammatory and fibrotic remodeling in atrial fibrillation. Epigenetic mechanisms—including DNA methylation, histone modification, and non-coding RNA signaling—have been implicated in the regulation of cytokine expression, fibroblast activation, and immune cell signaling within the atrial myocardium. These processes contribute to atrial cardiomyopathy and sustained inflammatory activation, thereby shaping both arrhythmia persistence and systemic inflammatory tone. Importantly, epigenetic dysregulation may also influence susceptibility to neuroinflammation by modulating endothelial function, blood–brain barrier integrity, and microglial responsiveness. This epigenetic–inflammatory axis provides a plausible biological link between atrial remodeling and downstream cerebral vulnerability, further supporting inflammation as a mechanistic mediator rather than a passive correlate in the AF–cognitive decline relationship [23].

### 3.4. Shared Risk Factors

A critical consideration when interpreting the AF–cognition association is the substantial overlap in risk factors for both conditions. Hypertension, diabetes mellitus, obesity, heart failure, obstructive sleep apnea, smoking, and physical inactivity are all established risk factors for AF and have also been independently linked to cognitive decline and dementia [6,24,25]. This overlap raises important questions about causality: does AF directly contribute to cognitive decline, or are both conditions downstream consequences of shared upstream risk factors? While most epidemiological studies attempt to adjust for these confounders, residual confounding remains a significant concern given the observational nature of the evidence.

## 4. Summary of Evidence

Table 1 summarizes key studies examining the association between AF and cognitive outcomes, including study characteristics, main findings, and quality assessments.

## 5. Preventive and Therapeutic Strategies

Interpretation of preventive and therapeutic strategies in atrial fibrillation requires careful distinction between levels of evidence and inferential strength, as summarized in Box 1.

Box 1 **Evidence–Inference Hierarchy Linking AF Management to Cognitive Outcomes.**

   Interpretation of preventive and therapeutic strategies in atrial fibrillation requires careful distinction between levels of evidence and inferential strength.

   Level I (high certainty): Randomized controlled trials provide robust evidence that oral anticoagulation reduces the risk of clinical ischemic stroke in patients with atrial fibrillation.

   Level II (moderate certainty): Neuroimaging studies demonstrate associations between AF management strategies and surrogate markers of brain injury, including silent cerebral infarction, white matter hyperintensities, and cerebral microbleeds.

   Level III (low–moderate certainty): Associations between AF therapies (e.g., anticoagulation, rhythm control, catheter ablation) and cognitive outcomes are derived predominantly from observational studies and remain hypothesis-generating.

   Consequently, while stroke prevention is an established benefit of AF management, potential effects on cognitive decline or dementia should be interpreted cautiously until confirmed by randomized trials with prespecified cognitive endpoints.

### 5.1. Oral Anticoagulation

Oral anticoagulation (OAC) is the cornerstone of stroke prevention in AF. The CHA_2_DS_2_-VASc score is widely used for stroke risk stratification [29,30,31]. Recent ESC 2024 guidelines have introduced the CHA_2_DS_2_-VA score, which omits the sex category to simplify decision-making [4]. Direct oral anticoagulants (DOACs) have largely replaced vitamin K antagonists (VKAs) as first-line therapy due to their favorable risk-benefit profile, with approximately 50% reduction in intracranial hemorrhage (RR 0.48; 95% CI: 0.39–0.59) [32].

Several observational studies have suggested that DOACs may be associated with lower dementia risk compared to VKAs. A large retrospective cohort study found DOACs associated with a 16% reduction in dementia risk (HR 0.84, 95% CI 0.72–0.99) compared to VKAs [26]. Proposed mechanisms include more stable anticoagulation intensity, reduced microbleeds, and potentially direct neuroprotective effects through thrombin pathway modulation [32]. However, these studies were observational and subject to confounding by indication—patients prescribed DOACs may differ systematically from those prescribed VKAs. No pivotal DOAC trials were designed with cognitive endpoints as primary outcomes [32].

### 5.2. Rhythm and Rate Control

Rhythm control strategies aim to restore and maintain sinus rhythm through catheter ablation or cardioversion. Evidence for the cognitive benefits of rhythm control is limited and inconsistent. While some observational studies suggest catheter ablation may be associated with reduced dementia risk (HR 0.56; 95% CI: 0.40–0.78 in one propensity-matched analysis), these studies are subject to substantial selection bias—patients selected for ablation tend to be younger, healthier, and more likely to achieve sustained sinus rhythm [28]. The EAST-AFNET 4 trial demonstrated cardiovascular benefits of early rhythm control (composite HR 0.79; 95% CI: 0.66–0.94) but did not include cognitive endpoints [33].

Rate control strategies, which aim to manage ventricular rate without restoring sinus rhythm, remain an acceptable alternative for patients with AF, though their effects on cognitive outcomes have not been systematically evaluated [34].

The CABANA trial compared catheter ablation with drug therapy for atrial fibrillation and demonstrated improved rhythm control and quality-of-life outcomes, though cognitive endpoints were not prespecified [35].

Although peri-procedural silent cerebral infarctions are frequently detected by diffusion-weighted MRI after catheter ablation, their relationship with long-term cognitive trajectories remains uncertain, with several studies reporting no consistent association between imaging findings and subsequent cognitive decline.

The AF burden hypothesis proposes that cumulative time spent in atrial fibrillation—rather than simply the presence or absence of AF—may be the critical determinant of cognitive risk. Observational studies demonstrate that AF is associated with worse cognitive outcomes and structural brain changes [11,36,37], and device-based studies have established dose–response relationships between AF burden and thromboembolic risk. Whether a similar burden-dependent effect exists for cognitive decline remains to be determined in prospective studies with continuous rhythm monitoring and longitudinal neuroimaging.

### 5.3. Left Atrial Appendage Occlusion

The left atrial appendage (LAA) is the source of cardioembolic thrombi in approximately 90% of cases of nonvalvular AF [38]. LAA occlusion (LAAO) has emerged as a potential alternative to chronic anticoagulation for patients with contraindications. Evidence from the PROTECT AF and PREVAIL trials demonstrated non-inferiority to warfarin for stroke prevention, though these trials did not assess cognitive outcomes [39].

### 5.4. Risk Factor Modification

Given the substantial overlap in risk factors for AF and cognitive decline, comprehensive risk factor management represents a logical strategy for preserving cognitive function. The ARREST-AF and LEGACY studies provided proof-of-concept that aggressive risk factor management—including weight loss, blood pressure control, glycemic control, OSA treatment, alcohol reduction, and smoking cessation—can reduce AF burden by up to 50% and improve arrhythmia-free survival [40,41]. Table 2 summarizes modifiable risk factors and recommended interventions.

## 6. Discussion

This narrative review aimed to synthesize current evidence linking atrial fibrillation with cognitive decline and to integrate epidemiological, mechanistic, and therapeutic data into a unified conceptual framework.

The evidence synthesized in this review supports reconceptualizing AF not merely as a cardiac rhythm disorder, but as a systemic vascular disease with important cerebral manifestations—a “cardio-cerebral syndrome.” Under this framework, AF represents both a consequence and a cause of vascular dysfunction: the same risk factors that promote AF (hypertension, diabetes, obesity, inflammation) simultaneously damage the cerebral vasculature, while AF itself amplifies cerebral injury through hypoperfusion, microembolism, and inflammatory cascades. This bidirectional relationship creates a vicious cycle wherein AF and cerebral small vessel disease potentiate each other’s progression. The clinical implication is that optimal management of AF requires attention not only to stroke prevention and symptom control, but also to the broader vascular milieu affecting brain health. Framing AF as a marker of systemic vascular brain disease may help clinicians appreciate why comprehensive risk factor modification—beyond anticoagulation alone—is essential for preserving cognitive function.

Cognitive phenotyping varied substantially across the studies included in this review, contributing to heterogeneity in reported associations. Cohorts employed a range of assessment approaches, including global screening instruments such as the Mini-Mental State Examination (MMSE) and Montreal Cognitive Assessment (MoCA), domain-specific neuropsychological batteries, and administrative or diagnostic coding for dementia outcomes. These methods differ in sensitivity to early cognitive change and in their ability to capture specific domains such as executive function or processing speed, which may be particularly relevant in vascular and AF-related cognitive impairment. Such variability limits direct cross-study comparability and may partly explain inconsistencies in effect size estimates.

This narrative review synthesizes current evidence on the relationship between AF and cognitive decline. The epidemiological association is well-established: multiple large cohort studies and meta-analyses consistently demonstrate that AF confers approximately 1.4- to 1.6-fold increased risk of cognitive impairment and dementia, independent of clinical stroke [1,3]. Multiple mechanisms plausibly link AF to cognitive decline, including thromboembolism (clinical and silent strokes), cerebral hypoperfusion, systemic inflammation, and shared risk factors.

Regarding interventions, oral anticoagulation—particularly with DOACs—is associated with reduced dementia risk in observational studies [26]. However, no randomized trials have been specifically powered to detect effects on cognitive outcomes. The evidence for rhythm control, rate control, and LAAO on cognitive preservation is even more limited, though the AF burden hypothesis and exploratory analyses from rhythm-control trials warrant further investigation in dedicated cognitive-outcome studies.

Several limitations affect the current evidence base. The predominance of observational designs limits causal inference. Heterogeneity in cognitive assessment tools complicates comparison across studies. Follow-up duration may be insufficient to capture a gradual cognitive decline. Reverse causation remains a concern—early cognitive impairment may affect AF detection or treatment adherence.

Future research should prioritize randomized controlled trials with cognitive function as prespecified endpoints. The BRAIN-AF trial (NCT02387229) is evaluating rivaroxaban versus aspirin with longitudinal cognitive performance as the primary endpoint, assessed using standardized neuropsychological testing. The GIRAF study (NCT01994265) is assessing the effects of edoxaban on cerebral microbleeds and other MRI-based markers of small-vessel disease, providing surrogate neuroimaging endpoints relevant to cognitive decline. These studies represent important steps toward establishing causal links between AF management strategies and brain health.

## 7. Limitations

This review has several limitations. As a narrative review rather than a systematic review with meta-analysis, the literature search and synthesis may be subject to selection bias. We restricted inclusion to English-language publications. Publication bias may affect the literature. The heterogeneity of cognitive assessment methods limited quantitative comparisons. Most included studies were observational with inherent risks of confounding and reverse causation. Finally, most studies were conducted in Western populations, potentially limiting generalizability.

## 8. Conclusions

This review demonstrates that atrial fibrillation is consistently associated with increased risk of cognitive decline and dementia through multiple interrelated pathophysiological mechanisms. The evidence supports conceptualizing AF as part of a broader cardio-cerebral syndrome, where cardiac and cerebrovascular pathology share common origins and mutually reinforce disease progression. Current evidence supports comprehensive AF management, including guideline-directed anticoagulation, appropriate rhythm or rate control, and aggressive modification of shared vascular risk factors. Clinicians should recognize AF not only as a cardiac rhythm disorder but also as a potential marker and mediator of cerebrovascular disease. Randomized trials with cognitive endpoints are needed to establish causality and identify optimal preventive strategies for preserving brain health in AF patients.

## Figures and Tables

**Table 1:** Summary of key studies on atrial fibrillation and cognitive outcomes.

Study	Design	N	Assessment	Key Findings	Quality
Koh 2022 [1]	Meta-analysis	2.8 M	Dementia Dx	AF: HR 1.39 (1.25–1.55) cognitive impairment; HR 1.36 (1.23–1.51) dementia	High
Diener 2019 [3]	Review	N/A	Multiple	AF independently associated with cognitive decline; mechanisms include SCI, hypoperfusion	High
Sagris 2024 [26]	Retrospective cohort	38,432	ICD codes	DOACs: HR 0.84 (0.72–0.99) dementia risk vs. VKAs	Moderate
Kim 2020 [27]	Retrospective cohort	262,611	Claims data	SBP 120–129 mmHg: HR 0.78 (0.69–0.89) dementia in AF patients	High
Kalantarian 2013 [11]	Review	N/A	MRI	SCI prevalence 22–40% in AF vs. 10–15% controls; OR 2.6 (1.9–3.5)	High
Bunch 2011 [28]	Retrospective cohort	37,908	ICD codes	Ablation: HR 0.56 (0.40–0.78) dementia vs. no ablation (propensity-matched)	Moderate

AF = atrial fibrillation; DOACs = direct oral anticoagulants; Dx = diagnosis; HR = hazard ratio; ICD = International Classification of Diseases; MRI = magnetic resonance imaging; N/A = not applicable; OR = odds ratio; SBP = systolic blood pressure; SCI = silent cerebral infarction; VKAs = vitamin K antagonists.

**Table 2:** Modifiable risk factors and management strategies.

Risk Factor	Mechanism	Intervention
Hypertension	Atrial remodeling, left ventricular hypertrophy, cerebrovascular disease, white matter hyperintensity progression	Intensive BP control (SBP 120–130 mmHg) associated with lower dementia risk in AF cohorts [27]; target per guideline recommendations
Diabetes mellitus	Oxidative stress, advanced glycation end-products, microvascular injury, brain insulin resistance	Optimal glycemic control (HbA1c < 7%); consider agents with cardiovascular benefit (SGLT2i, GLP-1RA)
Obesity	Systemic inflammation, hemodynamic stress, epicardial adipose tissue, associated OSA	Weight reduction of 10% or more associated with significant AF burden reduction [41]; structured lifestyle intervention
Obstructive sleep apnea	Intermittent hypoxia, intrathoracic pressure swings, autonomic dysfunction, systemic inflammation	CPAP therapy; associated with reduced AF recurrence after cardioversion and ablation
Alcohol consumption	Direct cardiotoxicity, autonomic effects, electrolyte disturbances, nutritional deficiency	Abstinence or moderation (1 or fewer standard drinks per day); complete abstinence may reduce AF recurrence
Smoking	Endothelial dysfunction, oxidative stress, sympathetic activation, prothrombotic state	Complete cessation; pharmacotherapy and behavioral support as needed
Physical inactivity	Reduced cardiorespiratory fitness, metabolic dysfunction, increased adiposity	Moderate-intensity exercise 150 min/week; cardiac rehabilitation where indicated
Heart failure	Reduced cardiac output, neurohormonal activation, atrial stretch, shared pathophysiology with AF	Guideline-directed medical therapy; consider CRT where indicated; optimize volume status

BP = blood pressure; CPAP = continuous positive airway pressure; CV = cardiovascular; GLP-1RA = glucagon-like peptide-1 receptor agonist; OSA = obstructive sleep apnea; SBP = systolic blood pressure; SGLT2i = sodium-glucose cotransporter-2 inhibitor; WMH = white matter hyperintensities.

## Data Availability

No new data were generated in this review.
